# Plant metabolic adaptation to nitrogen scarcity: the role of biological nitrification inhibitors

**DOI:** 10.1042/EBC20250049

**Published:** 2026-07-30

**Authors:** Jens Sigurd Agger Raabyemagle, Kasper Hinz, William Thomas Wajn, Tomas Laursen

**Affiliations:** Dynamic Metabolons Group, Section for Plant Biochemistry, Department for Plant and Environmental Sciences, University of Copenhagen, Copenhagen, Denmark

**Keywords:** Biological nitrification inhibition, plant adaptation, root exudation, specialized metabolites

## Abstract

In response to soil nitrogen (N) scarcity, plants adapt through physiological plasticity and metabolic strategies that secure and conserve N. Beyond root architecture, microbial symbioses, and allelopathic inhibition of competing plants, many plant species release biological nitrification inhibitors (BNIs) from the roots that slow the microbial oxidation of ammonium to nitrate, retaining N in the root zone and improving N uptake. The diversity of BNIs spans a broad spectrum of chemical properties with highly hydrophobic molecules (e.g., sorgoleone, zeanone, brachialactone) concentrated at root–particle interfaces, while more hydrophilic or amphipathic compounds (e.g., methyl 3-(4-hydroxyphenyl) propionate, syringic acid, 6-methoxy-2-benzoxazolinone) diffuse farther into the soil, supporting spatially distributed inhibition in soil. While some of these molecules have been known for decades, their mode of action remains elusive and possibly acts through multiple targets including inhibition of key enzymes involved in microbial nitrification, namely, ammonia monooxygenase and hydroxylamine oxidoreductase, whereas others potentially chelate metal cofactors or destabilize membranes. Major gaps remain in current BNI research: most biosynthetic pathways and exudation mechanisms are unresolved, and linking BNI trait to field performance is highly dependent on soil conditions, climate variations, and microbial communities. We outline a research agenda linking enzymology, genomics, and rhizosphere ecology to decode BNI function for future breeding, engineering, or bioproduction, toward low-nitrification cropping systems.

## Introduction

Plants are sessile organisms that must persist in environments where the availability of mineral nutrients is spatially and temporally heterogeneous. To thrive under nutrient limitation, plants have evolved a diverse repertoire of physiological, developmental, and metabolic adaptations. Root system plasticity enables foraging scarce resources [1], while biochemical strategies allow plants to both mobilize nutrients from soil and reduce competition with neighbors [2,3]. Among the essential macronutrients, nitrogen (N) is often the main growth limiting factor [4], and as a result, plants display particularly elaborate mechanisms to secure and conserve it.

**Figure 1:**
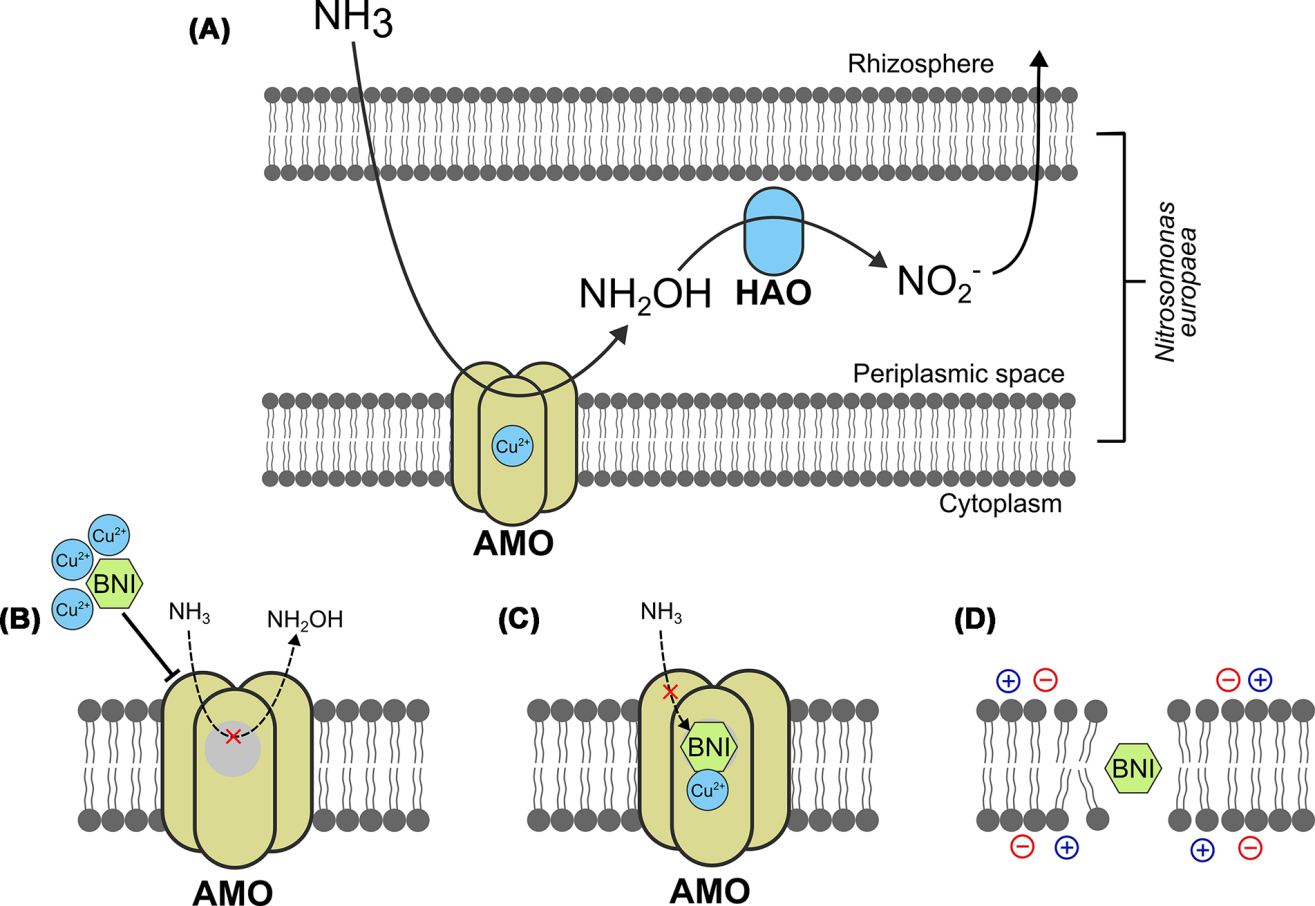
Plant strategies for nitrogen scarcity in soil environment Assitance-based (**A**) rhizobia–legume symbiotic relationship, where atmospheric N is fixated by rhizobia to produce ammonia for the plant in exchange for nutrients and shelter. Inhibition-based (**B**) allelopathic compounds are released by plants to inhibit neighboring plants. (**C**) BNIs are released by plants to inhibit nitrifying microbes to retain available N in the preferred form of ammonia. This also slows nitrate pollution and release of the greenhouse gas nitrous oxide (N_2_O).

Two overarching strategies stand out as evolutionary solutions for plants to adapt to nitrogen scarcity (Figure 1). First, assistance-based mechanisms involve mutualistic symbiosis with nitrogen-fixing microbes, most prominently the rhizobia–legume partnership [5] but also associations with actinobacteria [6] and cyanobacteria [7], allowing them to directly access atmospheric nitrogen. The former is the most well characterized, where leguminous plants such as *Vicia faba* (faba bean) employ bacteria belonging to the rhizobial group in root nodules to convert atmospheric nitrogen (N_2_) into the plant preferred source of N, ammonia (NH_3_), while in exchange providing carbohydrates and macronutrients to the rhizobia (Figure 1A). Second, inhibition-based strategies reduce competition and N loss through root-exuded secondary metabolites. Some plants produce allelopathic compounds in the roots that are released to the rhizosphere and suppress neighboring plant growth or germination and thereby reduce competition for available soil nitrogen and other resources. The root exudation of momilactones by *Oryza sativa* (rice) is a classic example, known for its strong weed-suppressing activity [3] (Figure 1B). Certain plant species have evolved the biochemical capacity to synthesize and exude another set of secondary metabolites, termed biological nitrification inhibitors (BNIs), which hinder soil microorganisms called nitrifiers from converting ammonia (NH_3_) into nitrate (NO_3_^−^)—a process known as nitrification (Figure 1C).

Nitrification represents a critical vulnerability in plant nitrogen management. Ammonia is retained in the root zone and can be readily assimilated, whereas nitrate is highly mobile and prone to leaching beyond the reach of roots as well as being lost through denitrification (Figure 1C), with estimates suggesting that up to half of available nitrogen in soils may be lost through these processes [8]. Thus, the evolution of BNIs can be viewed as a metabolic adaptation to nutrient limitation and loss; by releasing specialized metabolites that suppress the activity of ammonia- and nitrite-oxidizing bacteria and archaea, plants inhibit the conversion of ammonium to nitrate, thereby conserving nitrogen in a less mobile form and avoid loss of nitrogen by denitrification (Figure 1C) [9]. Since the first characterization of BNI activity in the tropical forage grass *Brachiaria humidicola*, originally found to exude substances from the roots with strong nitrification inhibition activity in soil [10], this trait has been documented across diverse lineages, including important cereals such as *Sorghum bicolor* (sorghum) [11], certain *Triticum aestivum* (wheat) landraces [9], *Zea mays* (maize) [12], and *O. sativa* (rice) [13]. The scale of the effect can be striking, with certain wheat landraces from N-scarce environments reducing soil nitrification rates by nearly 80% relative to unaffected rates [14]. These findings highlight BNIs as a widespread and ecologically significant adaptation, raising the question of what chemical strategies plants deploy to achieve this effect. The present review will therefore cover the current understandings of the diversity of BNIs in plants, as well as insights into their mechanisms of action, biosynthesis, and release into the environment.

In modern agriculture, nitrification also poses a major challenge for the environment and climate. In addition to depleting the nutrient pool for crops, nitrification and nitrate leaching are causing severe algal blooms and oxygen depletion in water systems (eutrophication) and thereby loss of aquatic life [15,16]. Furthermore, in anaerobic conditions nitrate is microbially reduced (denitrification) to nitrous oxide (N_2_O), a potent greenhouse gas with a heat-trapping capability 273 times higher than CO_2_ (Figure 1C) [17–19]. The present review addresses a key gap in the BNI field by synthesizing current understanding of biosynthesis, mode of action, and soil diffusion, while highlighting the research priorities needed to unlock the full potential of BNIs in modern agriculture.

## BNI diversity as an adaptive trait

**Figure 2:**
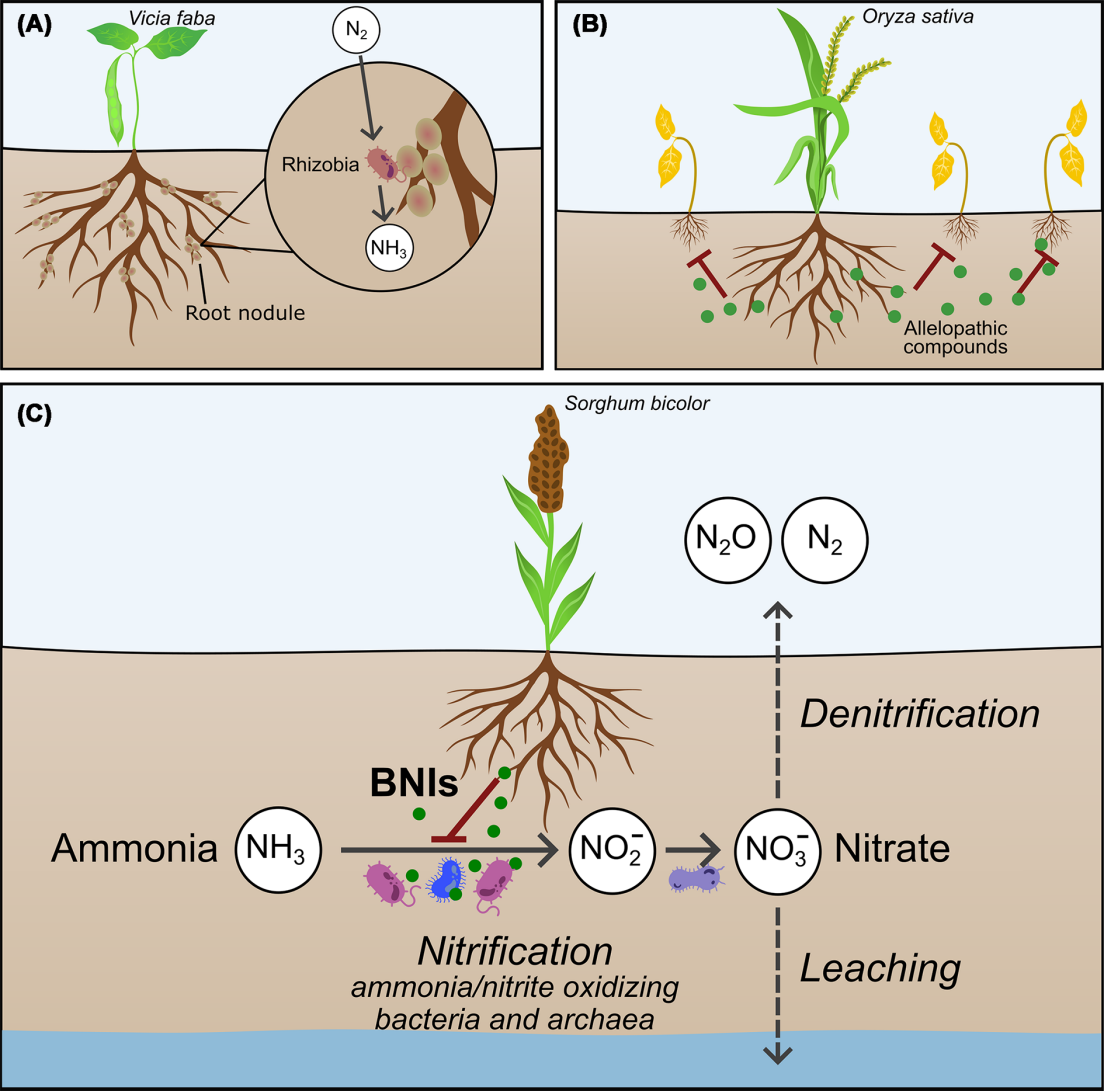
Chemical diversity and polarity of select BNIs Chemical diversity of select BNIs from (**A**) *Sorghum bicolor*, (**B**) *Brachiaria humidicola*, (**C**) *Zea mays*, and (**D**) *Oryza sativa*. Hydrophobicity of respective BNIs were calculated using the molecule’s raw logP (octanol–water partition coefficient) computed from its SMILES code and plotted on a logP axis from −2 (very hydrophilic) to 6 (very hydrophobic).

To date, only a handful of BNIs have been identified in plants, yet their chemical diversity is striking. They encompass multiple classes of specialized metabolites, including phenolics [20,21], flavonoids [22], quinones [23], fatty acids [13], indole-derived benzoxazinoids [12], and terpenoids [24], reflecting the diverse biosynthetic pathways that plants have evolved to mitigate nitrification in their rhizosphere. Importantly, this chemical variety is not random but suggests an adaptive logic: plants appear to deploy cocktails of BNIs with contrasting physicochemical properties to achieve spatially distributed inhibition across the rhizosphere. Hydrophobic BNIs such as sorgoleone in sorghum localize near root and membrane interfaces, where they may directly encounter nitrifiers attached to root surfaces or soil particles in close vicinity [11]. In addition to sorgoleone, sorghum produces a suite of BNI-active compounds including the phenolic compound methyl 3-(4-hydroxyphenyl) propionate (MHPP) [25] and the flavonoid sakuranetin (Figure 2A) [11]. Interestingly, the polarity of these compounds varies from sorgoleone, suggesting that BNIs exuded by sorghum diffuse through the rhizosphere and generate a concentration gradient from the root zone depending on the water solubility of the compounds. Sorgoleone, being very hydrophobic, might have evolved to function at the root–soil interfaces, while the less hydrophobic sakuranetin and hydrophilic MHPP will be more mobile in soil, mitigating nitrification in a broader rhizosphere. Maize employs a similar BNI gradient with the hydrophilic benzoxazinoids 2-hydroxy-4,7-dimethoxy-2H-1,4-benzoxazin-3(4H)-one and 6-methoxy-2-benzoxazolinone being complemented with the amphipathic naphthoquinone zeanone (Figure 2B) [12]. *Brachiaria humidicola*, one of the most BNI-active forage grasses, produces the diffusible phenolic aldehyde vanillin alongside the structurally distinct diterpenoid brachialactone (Figure 2C) [21,24]. The BNI capacity in *O. sativa* includes a combination of the phenolic syringic acid, highly soluble in water, and the hydrophobic fatty alcohol 1,9-decanediol (Figure 2D) [13,26]. Taken together, these examples suggest that plants have repeatedly converged on the strategy of combining inhibitors with a polarity gradient to optimize spatial suppression of nitrifiers in soils of different texture, moisture, and microbial density.

Variation among cultivars of the same species further supports this adaptive view. Sorghum genotypes differ widely in both the amount and composition of BNIs they exude, with some lines releasing markedly more sorgoleone or MHPP than others [11,27]. Wheat landraces likewise show strong differences in their root exudate profiles and nitrification suppression capacity, suggesting that the BNI trait is shaped by both natural selection and domestication [14]. This intraspecific diversity implies that the balance of hydrophilic and hydrophobic BNIs may be fine-tuned to local environments, from well-drained upland soils where diffusion dominates, to more compact or organic-rich soils where compounds may persist at particle surfaces [28].

This biochemical toolkit reflects an evolutionary adaptation to modulate soil nitrogen cycling through multiple spatial and mechanistic modes of action. The chemical diversity is encouraging, because it offers multiple avenues to explore in developing BNI-based solutions; at the same time, it presents a challenge for scientists to identify, characterize, and predict active compounds amid the complex mixture of plant exudates. Given the vast metabolic capacity of plants [29], it is plausible that many effective BNIs have yet to be identified, even within well-characterized crops. Untapped diversity in wild species and landraces will undoubtedly reveal novel inhibitors with unique modes of action. At the same time, the broader activity reported for some known BNIs [30–32] raises the possibility that some compounds may exert an unspecific general antimicrobial activity. Distinguishing true enzymatic inhibitors from nonspecific bioactive compounds remains a key challenge. Understanding how BNIs function at the molecular level is therefore critical not only to confirm their specificity, but also to enable their deployment in agroecosystems.

## BNI mode of action

**Figure 3:**
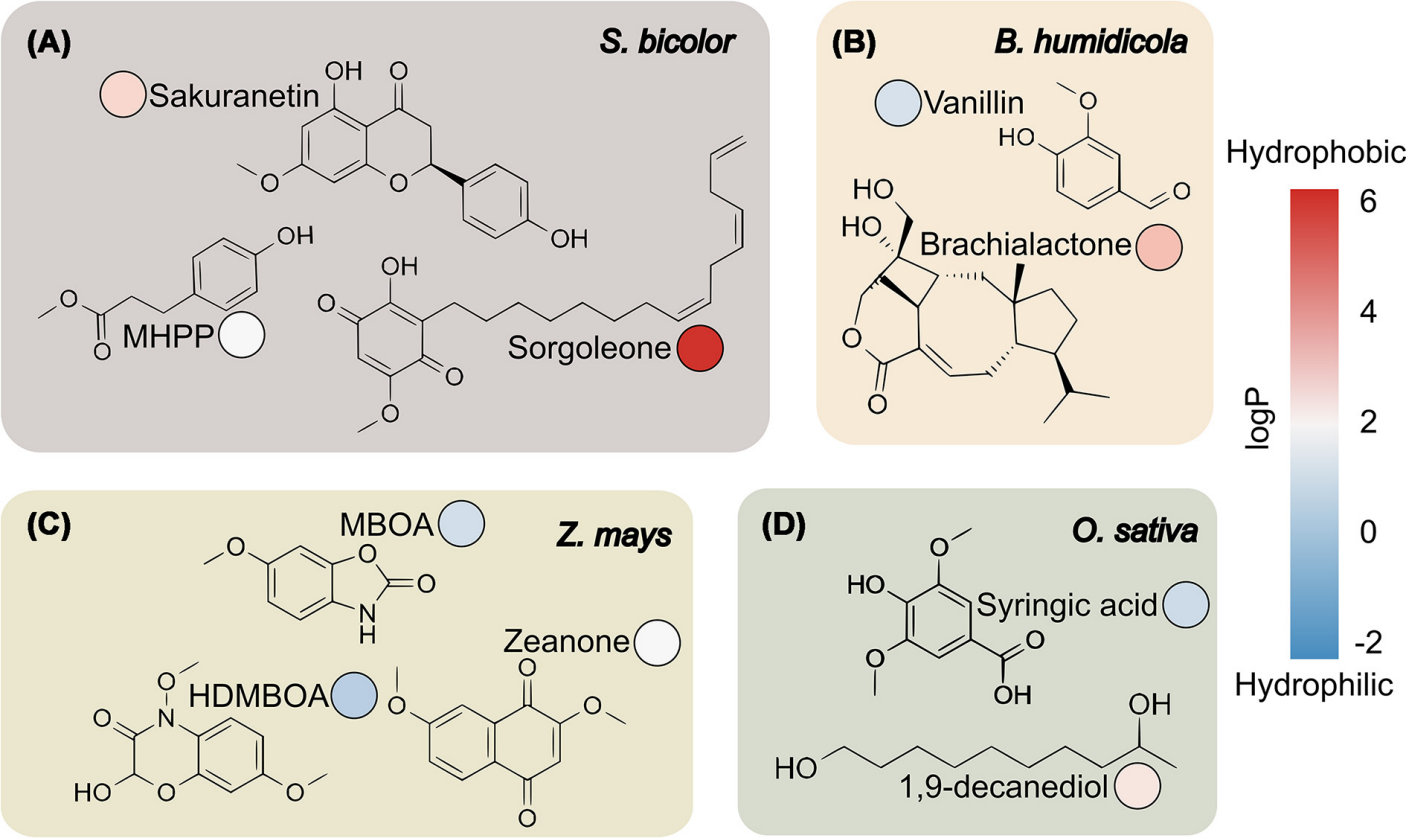
Putative BNI modes of action Putative BNI modes of action (**A**) First two steps of nitrification in AOB catalyzed by AMO and HAO. (**B**) Some BNIs are thought to chelate copper ions needed for AMO to function. (**C**) BNIs may function as irreversible inhibitors, in which covalent modifications of the enzyme will render it permanently inactive. (**D**) Antimicrobial effects such as membrane depolarization will interfere with essential cellular functions, thereby implicitly inhibiting enzyme function.

Although a growing number of plant-derived compounds have been identified as BNIs, the mechanistic understanding of how they interfere with microbial nitrification remains limited. Most of the current knowledge is derived from a combination of pure-culture experiments, soil microcosms, and indirect biochemical assays. Few BNIs have been characterized in detail, and the precise molecular targets and inhibition modes often remain speculative. To understand BNI activity, it is helpful to briefly outline the first steps of the microbial nitrification pathway. In ammonia-oxidizing bacteria such as *Nitrosomonas europaea*, ammonia is oxidized in a rate-limiting step to hydroxylamine (NH_2_OH) by ammonia monooxygenase (AMO), a copper-dependent metalloenzyme embedded in the cell membrane facing the periplasmic space [33,34]. Hydroxylamine is subsequently converted into nitrite (NO_2_^−^) by hydroxylamine oxidoreductase (HAO), a soluble periplasmic enzyme [35]. Thus, AMO and HAO have emerged as key targets for inhibition by BNI (Figure 3A).

Experimental evidence from *in vitro* culture and soil studies confirms this view, indicating that some BNIs can act directly on the key enzymes of ammonia oxidation, AMO, and HAO. For example, the *O. sativa* derived 1,9-decanediol has been linked to inhibition of the AMO-mediated oxidation of ammonium, resulting in ammonium accumulation in soils [13]. Other compounds, including sorgoleone, brachialactone, and certain fatty acids, have been reported to reduce activity of both AMO and HAO in *N. europaea* cultures [9,11,24,36]. These conclusions are generally based on *in vitro* culture assays using a single nitrifier and measuring substrate turnover rates or accumulation of pathway intermediates: reduced nitrite production or hydroxylamine build-up indicates specific enzymatic steps being blocked. Such results suggest that plants deploy BNIs not as indiscriminate antimicrobials, but as targeted tools for slowing nitrification where it matters most.

Nevertheless, the molecular mechanisms of inhibition remain largely unresolved. Some BNIs may act through metal chelation: the synthetic nitrification inhibitor 3,4-dimethylpyrazole phosphate is known to chelate copper and is hypothesized to thereby block AMO activity by either chelating all available copper or by interaction with copper in the AMO active site [37,38]. Plant phenolics such as quercetin and rutin possess similar metal-binding properties [39], raising the hypothesis that certain BNIs act by depriving AMO of essential cofactors (Figure 3B) [9,24]. AMO is known to undergo mechanism-based (‘suicide’) inactivation when it oxidizes certain alternative substrates such as acetylene or allylsulfide, producing reactive intermediates that irreversibly inactivate the enzyme and require *de novo* protein synthesis for recovery [35,40]. By analogy, it has been hypothesized that some plant-derived BNIs may act in a similar fashion, being oxidized by AMO into reactive products that block activity (Figure 3C), although direct biochemical evidence for such suicide inhibition by BNIs is still lacking [9,41,42]. It is also important to consider that some BNIs may act through broader antimicrobial mechanisms. Several compounds, such as vanillin and syringic acid, have been shown to de- or hyperpolarize microbial membranes [43], reduce intracellular pH [44], or impair respiratory function [31], raising the question of whether their inhibitory effects on nitrification stem from specific enzymatic targeting or general microbial toxicity (Figure 2D). From an adaptive perspective, this distinction is critical. Specific inhibition of nitrifiers would maximize nitrogen conservation with minimal disruption of the wider microbiome, while broad-spectrum toxicity could undermine beneficial microbial associations.

Many BNIs also retain additional ecological functions, underscoring their multifunctionality as plant metabolites. Sorgoleone doubles as a potent allelochemical that disrupts photosynthesis in competitor seedlings [45,46], sakuranetin acts as an antifungal defense compound [47], and benzoxazinoids in maize shape entire microbial communities beyond nitrifiers [48]. This overlap between defense chemistry and rhizosphere nitrogen management highlights that BNI activity may often be layered onto existing metabolic pathways, co-opted as part of plants’ adaptive repertoire to thrive under nitrogen-limited conditions. Future research should focus on in-depth characterization of the mode of action linking biochemistry, microbiology, and soil-nitrogen cycling studies. It will be important to link the BNI potential with binding and inhibition studies of AMO and HAO, ideally through purified enzyme systems. The specificity should be thoroughly assessed through quantitative microbiome profiling evaluating the growth inhibiting effects on all nitrifiers versus the global microbial communities. Importantly, some compounds like sakuranetin have been shown to have high BNI activity *in vitro* but display limited activity in soil-based experiments [11], underscoring the need to also bridge controlled assays with environmentally relevant conditions. The in-depth analysis of BNI compounds and link to chemical properties will enable genomics approaches to identify genetic traits governing the BNI trait.

## Biosynthesis and exudation of BNIs

Recent focus on identification of BNI compounds in relevant crop wild relatives has demonstrated an impressive chemical diversity. However, the molecular basis of BNI biosynthesis and secretion remains poorly understood. For a few compounds, partial or complete pathways have been elucidated, offering essential insights into the adaptation of plant specialized metabolism to conserve nitrogen under limiting conditions.

Sorgoleone (Figure 2A), for example, is a lipid-derived metabolite that is activated via a polyketide pathway in root hair cells [32], by the action of several desaturases [49], alkylresorcinol synthases [50], an O-methyltransferase [51], and a cytochrome P450 enzyme [52]. However, the upstream fatty acid release and CoA-activation step supplying the alkylresorcinol synthases remains unidentified [53]. Moreover, sorgoleone is released from root hairs in a reduced dihydroquinone state that undergoes oxidation in the rhizosphere to yield the fully active benzoquinone form [52], underscoring that some BNIs are not chemically matured within the plant but complete their activation in soil. The biosynthetic pathway of sakuranetin has been elucidated in *O. sativa*, branching from flavonoid production [54], with the final step catalyzed by an O-methyltransferase that converts naringenin into sakuranetin [22]. Syringic acid in *O. sativa* and vanillin in *B. humidicola* have both been identified as BNIs, with well-characterized biosynthetic pathways, owing to the pharmacological relevance of syringic acid [55] and vanillin’s role as an aroma compound [56], where both are known to derive from the phenylpropanoid pathway via shikimate-origin intermediates. For most other BNIs, including brachialactone, MHPP, and related phenolics, the complete biosynthetic pathways have yet to be resolved, underscoring a major knowledge gap between identifying bioactive exudates and understanding their enzymatic origin.

The adaptive function of BNIs depends on their efficient secretion into the soil; however, the molecular mechanisms that mediate secretion are largely unknown. Sorgoleone has been localized to root hair vesicles and secreted via a lipid-droplet-like mechanism [57], suggesting a highly specialized system for packaging and export of highly hydrophobic molecules. For most other BNIs, the transport processes are completely unknown. Whether specialized membrane transporters are involved, as in the case of some plant specialized metabolites [58], has yet to be demonstrated.

These gaps in knowledge highlight a major frontier in understanding BNI adaptation. To advance our understanding of plant adaptation to nitrogen limitation and realize the full potential of BNI as a future crop trait, we need to identify the structural genes and transport mechanisms that underlie biosynthesis and exudation.

## Challenges and future directions

Since the Green Revolution, fertilizer application has risen dramatically [59], yet crop recovery rarely exceeds 50%, with the remaining nitrogen lost via leaching or gaseous emissions [8], causing severe environmental harm due to the high amounts of nitrate generated. In line with the degradation of agricultural soil and increasing attention on sustainable agricultural practices, it has become highly attractive to realize the potential of biological solutions to improve nitrogen-use efficiency and reduce the input requirements. Thus, leveraging the natural potential of some plants to produce and secrete bioactive compounds with the capacity to reduce microbial nitrification has been suggested as a key strategy. This depends on a fundamental understanding of the BNI mode of action, their biosynthetic pathways, and transport mechanisms.

In natural systems, BNIs allow plants to retain nitrogen in the less mobile ammonium form, improving competitiveness under nutrient-poor conditions. In modern agriculture, the same principle could help address one of the most pressing challenges: the inefficiency of synthetic nitrogen fertilizer use. Several avenues are emerging. Research initiatives are currently looking into conventional breeding and marker-assisted selection that offer routes to introgress BNI traits from wild relatives of wheat into elite cultivars [60–62]. Advances in molecular biology and synthetic biology may further enable direct engineering of BNI pathways or transport processes into crops with otherwise weak inhibitory capacity. Another possibility is the biotechnological production of purified BNIs for use as soil amendments, either alone or in combination with organic fertilizers. However, these applications are currently constrained by major knowledge gaps: the biosynthetic pathways of many BNIs remain unresolved, exudation mechanisms are poorly understood, and field-level performance is highly variable across soils, climates, and microbial communities [63].

Bridging these gaps requires interdisciplinary research linking structural biology of enzyme targets, comparative genomics of biosynthetic genes, and field ecology of rhizosphere processes. By integrating BNIs into agricultural practice, through breeding, biotechnology, or sustainable soil management, plants’ evolutionary strategies for nitrogen conservation can be repurposed as practical tools for climate-smart agriculture. In this way, BNIs exemplify how insights into plant adaptation can inform solutions to one of agriculture’s greatest sustainability challenges.

## Summary

- Plants adapt to nitrogen scarcity through BNIs that conserve ammonium in the rhizosphere by inhibiting microbial competitors for ammonia.
- BNIs are chemically diverse and often secreted as a cocktail of metabolites with ranging polarity, enabling spatially distributed inhibition of nitrifiers across soil environments.
- Evidence suggests that BNIs can act through multiple mechanisms, including direct inhibition of the rate-limiting nitrification enzymes, metal chelation, or broader antimicrobial effects.
- Knowledge of biosynthetic and exudation pathways remains limited; only a few compounds have partially resolved pathways.
- Understanding BNIs as an adaptive plant trait provides a basis for translating them into agriculture through breeding, engineering, or biotechnological production, to improve nitrogen-use efficiency and reduce fertilizer losses.

## Open Access

Open access for this article was enabled by the participation of the University of Copenhagen in an all-inclusive Read & Publish agreement with Portland Press and the Biochemical Society under an agreement with the Royal Danish Library.

## Abbreviations

AMO: ammonia monooxygenase
AOB: ammonia-oxidizing bacteria
BNI: biological nitrification inhibitor
HAO: hydroxylamine oxidoreductase
MHPP: methyl 3-(4-hydroxyphenyl) propionate
N: nitrogen
SMILES: Simplified Molecular Input Line Entry System
CoA: Coenzyme A
